# EEG-Based BCI System to Detect Fingers Movements

**DOI:** 10.3390/brainsci10120965

**Published:** 2020-12-10

**Authors:** Sofien Gannouni, Kais Belwafi, Hatim Aboalsamh, Ziyad AlSamhan, Basel Alebdi, Yousef Almassad, Homoud Alobaedallah

**Affiliations:** College of Computer and Information Sciences, King Saud University, Riyadh 12372, Saudi Arabia; gnnosf@ksu.edu.sa (S.G.); hatim@ksu.edu.sa (H.A.); ZAlSamhan@ksu.edu.sa (Z.A.); BAlebdi@ksu.edu.sa (B.A.); YAlmassad@ksu.edu.sa (Y.A.); HAlobaedallah@ksu.edu.sa (H.A.)

**Keywords:** EEG, brain-computer interface, prosthetic finger, decoding finger movement, multi-class classification

## Abstract

The advancement of assistive technologies toward the restoration of the mobility of paralyzed and/or amputated limbs will go a long way. Herein, we propose a system that adopts the brain-computer interface technology to control prosthetic fingers with the use of brain signals. To predict the movements of each finger, complex electroencephalogram (EEG) signal processing algorithms should be applied to remove the outliers, extract features, and be able to handle separately the five human fingers. The proposed method deals with a multi-class classification problem. Our machine learning strategy to solve this problem is built on an ensemble of one-class classifiers, each of which is dedicated to the prediction of the intention to move a specific finger. Regions of the brain that are sensitive to the movements of the fingers are identified and located. The average accuracy of the proposed EEG signal processing chain reached 81% for five subjects. Unlike the majority of existing prototypes that allow only one single finger to be controlled and only one movement to be performed at a time, the system proposed will enable multiple fingers to perform movements simultaneously. Although the proposed system classifies five tasks, the obtained accuracy is too high compared with a binary classification system. The proposed system contributes to the advancement of a novel prosthetic solution that allows people with severe disabilities to perform daily tasks in an easy manner.

## 1. Introduction

In 2006, a “Convention on the Rights of Persons with Disabilities” (UNCPRD) was adopted by the United Nations that recognizes the autonomy and independence of living as basic human rights [1]. In line with this treaty, the system proposed in this paper aims to help people with severe disabilities restore the mobility of their fingers using Brain Computer Interface (BCI) technology. BCI technology aims to assist people suffering from severe disabilities in regular everyday activities by proposing an affordable alternative pathway to regain communication and interaction with their environment. Over the past decade, the BCI technology has seen a rapid development and it has been applied to a wide range of fields, especially in rehabilitation engineering or for functional substitution [2,3,4,5,6].

Different types of bio-signals such as EEG [7,8,9,10], Magnetoencephalography (MEG) [11,12], Electrocorticogram (ECoG) [13,14,15], Functional Near-Infrared Spectroscopy (fNIRS) [16], as well as Electromyography (EMG) [17] have been used to design BCI systems that decode finger movements. EEG is the most commonly used technology for the acquisition of brain signals in BCI systems due to its noninvasive nature, low cost, and high portability [8]. EEG signals corresponding to motor imagery (MI) are defined in frequency domains as brain activity that is triggered by muscular contractions or by thinking about specific tasks. Sensorimotor rhythms (SMRs) activity are registered during motor imagery and they allow the design of the so-called motor-imagery BCI (MI BCI). SMRs appear in well-defined locations according to a well known functional map that binds SMRs with the brain lobes where they took place [18].

Distinct brain activity patterns related to the five fingers’ movements should be identified to control prostheses. The discrimination of finger movements is a complex task due to physiological and non-physiological artifacts in the EEG signals [19]. Unlike movements performed by different parts of the human body, the movements of different fingers of the same hand activate relatively small and close regions in the sensory-motor cortex area [8]. Furthermore, an advanced classification strategy that addresses multi-class classification problems is required by such applications. Therefore, all artifacts must be carefully removed and the signal-to-noise ratio must be enhanced, as well as the relevant channels containing the useful and non-redundant information must be selected, and an appropriate classification strategy must be applied to discriminate between different tasks with a high degree of accuracy.

The vast majority of existing EEG-based BCI systems analyze brain activities to identify a single finger movement. These systems are unreliable to control prostheses and robotic hands that are designed for performing tasks that require various skills [8]. Ref. [7] analyzed Event-related desynchronization (ERD) topography during left or right index finger movement. A degree feature extraction algorithm was proposed based on the graph theory together with Support Vector Machine (SVM) to classify two kinds of index finger movement: left or right index finger movement. The average accuracy of the system was about 62%. Ref. [8] proposed decoding finger movements, including four thumb-related movements as well as the flexion and extension movements of the index, middle, ring, and little fingers. The system predicts the movements using the Choi-Williams distribution and a two-layer classification framework. The system obtained an average classification accuracy computed over the four fingers across all subjects of 43.5%. Ref. [9] proposed another system that applies the common spatial pattern (CSP) algorithm to extract features, as well as four classifiers, such as the random forest, SVM, k-nearest neighborhood (kNN), and the linear discriminant analysis (LDA) to discriminate between trials. The maximum average accuracy reached by these classifiers is about 54%. Ref. [10] proposed using the principal component analysis (PCA), the Power Spectral Densities (PSD), and SVM with a Radial Basis Kernel Function (RBF). The average accuracy of the system was about 77%.

Nevertheless, a few BCI systems have been proposed in the literature to decode the movements performed by each finger within the same hand [10] and the wrist/grasp-related movements of the same hand [8,20]. Recently, an investigation has been started to develop new algorithms allowing researchers to decode the simultaneous movements of the five fingers.

Previous EEG-based studies [7,8,9,10] were usually extracting features from the same group of electrodes for all subjects and for all fingers movements, whereas neuroscience studies mentioned that the activity is unique in the brain of each person. Moreover, the brain activity is different in the left and right brain hemispheres during the same motor imagery [7,21]. Thus, there is a growing need to analyze the brain activity during finger movements and to identify the electrodes that are showing relevant and significant changes. We present a novel statistical-based approach to identify, for each subject, electrodes that are relevant to motor imagery tasks of each finger. The selected electrodes were used as sources to extract relevant features. The experimental results show that the proposed method is highly competitive compared with the existing studies on a multi-class fingers movements classification. Five motor imagery tasks are considered in this work, including movements of the thumb, index, middle, ring, and pinky fingers.

The remainder of this paper is organized as follows. In Section 2, the methodology is presented and the EEG signal acquisition as well as the signal processing chain are described. In Section 3, experimental results are presented. Section 4 discusses the results, a conclusion is drawn, and a future work is indicated.

## 2. Materials and Methods

The experiments carried out in this paper were approved by the Targeted Research Program committee—Disability Research Grant Program at King Abdulaziz City for Science and Technology (KACST) which granted funding for the research project No 5-18-03-001-0015-10827.

**Figure 1:**
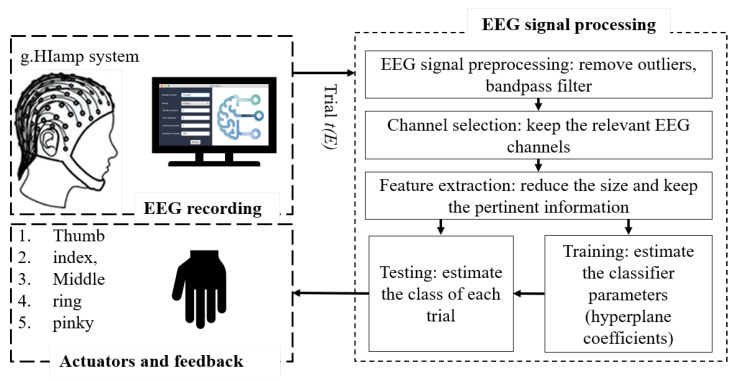
General structure of the proposed system.

Our methodology focuses on the requirements of a multi-class classification problem for a successful BCI system that decodes the movement of fingers using EEG brain activity signals. Figure 1 presents the general structure of the proposed system. Firstly, our own dataset was created using, a g.HIamp from g.tec, an 80 channel amplifier. To do this, volunteers were asked to randomly move their fingers. Every single finger movement was considered to be a single trial. EEG signals that correspond to the trials of the subject were recorded during different sessions. Every trial was labeled distinctively. Afterwards, the recorded data was processed by removing the artifacts to increase the signal-to-noise ratio (SNR) of the EEG signals. Subsequently, the set of electrodes showing signals that are relevant to the movements of the fingers was identified. Then, the CSP algorithm was applied to extract the features that correspond to the finger movements. Finally, an ensemble of one-class classifiers was used to decode the five finger movements. Every one-class classifier was trained to detect the movements of a given finger. To avoid over-fitting, every classifier was trained on a portion of the data set and tested using another portion. The measurement accuracy was used to determine the performance of every one-class classifier.

### 2.1. Data Acquisition

#### 2.1.1. EEG Signal Acquisition

The EEG signals were recorded using a g.tec g.HIamp amplifier. The signals were captured through 64 electrodes placed on the scalp according to the international system localization 10–20. 256 Hz was decided as the sampling frequency of the signals and a filtration stage was applied using a BPF with the type “Chebyshev” to keep the frequency component between 1 Hz and 60 Hz.

#### 2.1.2. Experimental Paradigm

The data used in this project was created locally and consisted of EEG signals recorded from 5 KSU volunteers aged between 21 and 23 years. All subjects were male and all of them were right-handed. As for the experiment, subjects were told to sit on a comfortable chair and their right arm was placed on a table to rest and thus to avoid muscle fatigue. We recorded EEG signals from every subject in multiple sessions. During every session, the selected subjects were asked to perform individual finger movements according to the automated scenario program interface (SPI). The subjects performed actions that corresponded to the movement of the five fingers and they were told to use only their right hand. All movements started at a neutral position, with the hand open, the lower arm extended to around 120 degrees, and the thumb placed on the inner side. Subjects were requested during every trial to execute sustained movements. For each finger, 180 trials were recorded which were comprised of 3 to 4 different sessions.

#### 2.1.3. Monitoring the Recording Sessions

Furthermore, a SPI was developed to guide the subjects during the recording of the sessions. It was responsible for displaying the instructions for the subjects on a screen. The main menu of the SPI and indicates the general information related to each subject, the scenario mode, the duration of readiness, the duration of flexing, the duration of waiting, and the number of trials in each session. Once the EEG recording process was launched, a specific state machine was followed; which included 3 phases:

- Get ready phase: during this phase, a random finger/limb movement was selected and the corresponding animated picture “gif file” was displayed by the scenario program on the screen.
- Action phase: during this phase, the subject moved the selected finger/limb.
- Rest phase: during this phase, the subject was in a resting state.

The duration of each recording session was as follows. 2 s were set for each phase; the get ready phase, the action phase, and the reading phase.

#### 2.1.4. Labeling Signals

The recording process was done over four sessions. Each session was continuous and was discretized into a sequence of six seconds per trial. The first 20 s of each session were discarded due to the BCI amplifier initialization delay. The EEG signals were subdivided into six-second intervals where each epoch corresponded to one finger movement. Each interval was labeled with a corresponding label from the scenario program. This process was repeated for every session and the processed sessions were concatenated to give each subject a fully labeled signal.

### 2.2. Artifacts Removal

After labeling the EEG signals, a filter block was applied to remove artifacts. In this way, only the frequency components related to the intention of finger movement were kept. These frequency components were often set to be between 8 Hz and 30 Hz [22]. Thus, a finite impulse response filter was applied with a 4th order, allowing the removal of frequency components outside the band while maintaining a zero-frequency phase for the signal [23]. Subsequently, a common average referencing technique was applied to allow the average signal at all electrodes to be calculated and subtracted from the EEG signal at every electrode for each time point. This step allowed for the discrimination between the positive and the negative peaks in the EEG signals and for locating the sources of the signal in the noisy environment that led to an improvement in the SNR [24]. The EEG signals were converted and computed according to Equation (1).

(1) $$T_{CAR}\left(n\right)=T\left(n\right)-\frac{1}{∥E∥}\sum_{k=1}^{∥E∥}T\left(k\right)$$

where ∥E∥ is the total number of electrodes used in the recording process of one trial *T*. T(k) is the EEG signal at the electrode *k*.

### 2.3. Selection of Relevant Electrodes

Unlike existing EEG-based studies to detect fingers movements which are extracting features from a predefined set of electrodes, this work proposes an adaptive method to analyze the brain activity during fingers movements and to identify the electrodes that are showing relevant changes. The identified electrodes were used as signal resources to extract relevant features before the classification step.

#### 2.3.1. Annotations

- An electrode (*e*) is an electrical conductor used to acquire brain signals.
- *E* is the set of electrodes (*e*) on a cap.
- A motor imagery (*m*), also called the motor imagery task, is a mental process by which an individual simulates a given movement action.
- Ψ is a set of motor imagery tasks. The motor imagery tasks which were considered here are the imaginary movements of the thumb, index, middle, ring and pinky fingers.
- A trial (*t*) is a set of brain signals that are recorded with a set of electrodes *E* during a given motor imagery task *m*.
- θ is a set of trials. θ= $\left\{t_{i}\right\}$
- ς is a set of subjects from each of which a set of trials was recorded.
- A rest is a set of brain signals, recorded using a set of electrodes *E* that corresponds to the mental state during a resting period. In this study, the resting period corresponds to the portion of a trial *t* recorded during the 0–1 s period of *t*. It is denoted t[0−1].

#### 2.3.2. Preliminaries

The following set of basic functions are required for the selection of the relevant electrodes:

- σ: For a given motor imagery task $m_{i}$, this function returns the subset of trials that were recorded during $m_{i}$. It is defined as follows:
  – σ: Ψ→P(θ)
  – $\sigma \left(m_{i}\right)=\{t_{j}\in \theta$ in a way that $t_{j}$ is recorded during the motor imagery task $m_{i}\}$.
- δ: For a given subject $s_{i}$, this function returns the subset of trials that have been recorded during the sessions of the subject $s_{i}$. It is defined as follows:
  – δ:ς→P(θ)
  – $\delta \left(S_{i}\right)=\{t_{j}\in \theta$ in a way that $t_{j}$ is recorded during a session of the subject $S_{i}\}$.
  – Pow(e,t) is the power, also called the energy, of the electrode *e* calculated from the trial *t*. It is computed using a spectral representation of the trial *t* with the application of the fast Fourier transformation. It is measured according to the following expression:
    (2) $$Pow\left(e,t\right)=\sum {}_{f\in [8,30]}f*FFT\left(t\right)\left[e,f\right]$$
    where
    (3) $$FFT\left[e,f\right]=\sum_{n=0}^{N-1}t\left[n,e\right]e^{\frac{-j2\pi kn}{N}}$$
    The power spectrum of each trial using the FFT function are available online at Supplementary Materials (Folder S1).
  – ERD/ERS(e,t,ref_Power) is defined as the percentage of the power increase or decrease in the electrode *e* during the trial *t* in relation to a reference power ref_Power, according to the following expression:
    (4) $$ERD\_ERS\left(e,t,ref\_Power\right)=\frac{Pow(e,t)-ref\_Power}{ref\_Power}$$

#### 2.3.3. The Selection Models

Let’s define the following functions:

- The function $\phi (s_{i},m_{j})$ that calculates the subset of trials recorded during the sessions of the subject $s_{i}$ while performing the motor imagery task $m_{j}$. It is defined as follows:
  – ϕ: Ψ→P(θ)
  – $\phi \left(s_{i},m_{j}\right)=\delta \left(s_{i}\right)\cap \sigma \left(m_{j}\right)$
- The function $\tau (s_{i},m_{j},e_{k})$ that calculates the subset of $\phi (s_{i},m_{j})$, where changes in the brain activity of the subject $s_{i}$ in the electrode $e_{k}$ are significant during the motor imagery task $m_{j}$. Brain activity variations during a given motor imagery are considered significant if they exceed the variation in power in a reference electrode during the same motor imagery. It is defined as follows:
  – τ: ς×Ψ×E→P(θ)
  – $\tau \left(s_{i},m_{j},e_{k}\right)=\{t_{l}\in \phi \left(s_{i},m_{j}\right)$ such that $|ERD\_ERS(e_{k},t_{l},ref\_Power\left(s_{i},e_{k}\right)|\geq |ERD\_ERS(reference_{elec},t_{l},ref\_Power\left(S_{i},reference_{elec}\right)|$
    * Where ref_Power ($s_{i}$, e) is defined as the average power of the electrode *e* during the distinct rest periods of $s_{i}$, according to the following expression:
      (5) $$ref\_Power\left(s_{i},e\right)=\frac{1}{∥\delta (s_{i})∥}\sum {}_{t\_x\in \delta (s\_i)}Pow\left(e,t\_x\left[0..1\right]\right)$$
    * Regarding the reference electrode ($reference_{elec}$) we selected $C_{3}$ as the reference for all electrodes located at the left-brain hemisphere. Moreover, we selected $C_{4}$ as the reference for all electrodes located at the right brain hemisphere.

A recent study published in Scientific Reports on 2020 [21] has presented the distribution of sensorimotor rhythms during hand motor imagery in the right and the left hemispheres. These distributions confirm neuroscience assumptions saying that brain activity corresponding to left hand movements is located on the right hemisphere and vice versa. This is why we considered the electrodes C3 and C4 as references to the electrodes at the left and right hemispheres respectively.

Take for instance a subject $s_{i}$, a motor imagery $m_{j}$ and an electrode $e_{k}$. The electrode $e_{k}$ is relevant to the motor imagery $m_{j}$ with respect to the subject $s_{i}$, if the probability, denoted $\rho (s_{i},m_{j},e_{k})$, of obtaining significant brain activity changes in $e_{k}$ when the subject $s_{i}$ performs the motor imagery task $m_{j}$ exceeds a given threshold called the α_threshold. The probability $\rho (s_{i},m_{j},e_{k})$ is calculated as follows:

- ρ:ς×Ψ×E→[0,1]
- $\rho \left(s_{i},m_{j},e_{k}\right)=\frac{∥\tau (s_{i},m_{j},e_{k})∥}{∥\phi (si,m_{j})∥}$

Here:

- $∥\tau (s_{i},m_{j},e_{k})∥$ and $∥\phi (si,m_{j})∥$ are the total number of trials of $\tau (s_{i},m_{j},e_{k})$ and $\phi (s_{i},m_{j})$, respectively.

Based on the function specifications described above, the following electrodes selector, called the ϵ, calculates and returns the set of electrodes that are relevant, for a given subject $s_{i}$, to a given motor imagery $m_{j}$. It is defined according to the following expression:

- ϵ:ς×Ψ→P(E)
- $\epsilon \left(s_{i},m_{j}\right)=\{E_{k}\in E$ such that $\rho (s_{i},m_{j},e_{k})\geq \alpha \_threshold\}$

### 2.4. Feature Extraction

For every finger movement *m* of a given subject *s*, we identified a set of relevant electrodes. These electrodes were used to extract appropriate features using the CSP method that is a well-known feature extraction technique. This method aims to extract and keep a significant activity or rhythm, as well as eliminate all redundant EEG signals. These features represent the most significant energy at the relevant electrodes in the α and β bands in reality; which have the highest likelihood of containing significant motor imagery information [22]. More theoretical details about the CSP algorithm are presented in [25].

### 2.5. Finger Movements Classification

For every Finger movement *m*, a one-class classifier denoted $C_{m}$ was designed. The classifier $C_{m}$ is responsible for the detection of the movements of the corresponding finger. The classification model of a given classifier $C_{m}$ is built including features extracted by the CSP algorithm using the subset of electrodes that are relevant to the corresponding motor imagery *m*. As such, each finger had its classification model, where the signal was copied to the model of each finger as well as each model was classified based on whether that signal belonged to that particular finger. This approach made the movement of multiple fingers at the same time possible, as each finger worked independently.

Many classifiers were tested, such as SVM, Logistic Regression, Gaussian Naive Bayes, and LDA. LDA was more efficient in term of accuracy against the other classifiers. Therefore, the LDA was selected as the main classifier of the proposed approach.

### 2.6. Prosthesis Control

The main objective of this work is to control prostheses merely by EEG motor imagery signals. A complex signal pre-processing is performed on EEG trials to remove artifacts, enhance signal to noise ratio, and decrease the dimensionality of the EEG signals. Every trial is processed simultaneously by each one-class classifier. Every one-class classifier applies the CSP algorithm on the set of electrodes that are relevant to the corresponding finger, to extract relevant features. Based on the extracted features, the LDA classifier identifies the trial as a target or as an outlier. If a given one-class classifier has recognized the trial as target, then a control command is sent to the corresponding finger’s actuator. A finite state machine corresponding to these steps is designed and validated at the simulation stage.

## 3. Results

The extracted features and their corresponding labels were split into two sets. The first set was dedicated to the training session and the other for testing purposes. The presented results were measured according to a 5-fold cross-validation approach. The training set for each finger was concatenated with 20% of the data from other fingers. However, for the testing set, 50% of the data from other fingers was added. Algorithm 1 summarizes the data set decomposition process.

**Figure 2:**
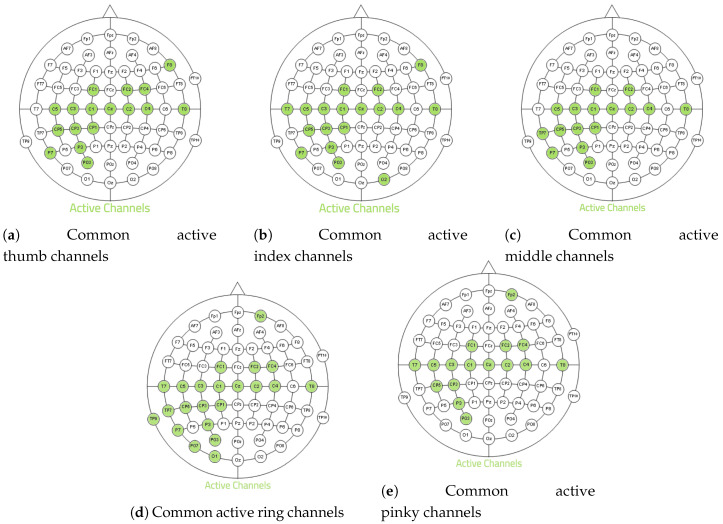
Relevant Channels for each finger.

The main objective of this section is to demonstrate the efficiency of the proposed method in regard to distinguishing the movements of the five-finger based on the intention of the user. Until now, the recording trials were applied directly to the signal processing chain without the application of the proposed channel selection algorithm. This is the first approach where the EEG features are extracted using the CSP spatial filter and the LDA algorithm for classification, while maintaining the same data partition as presented in Algorithm 1. The accuracy of the system fluctuates between subjects from 52% to 60%, with an average of 57%. This accuracy is poor, which makes the system inefficient. With the application of the same techniques and the integration of the channel selection method, the number of channels used during the acquisition are minimized by identifying the relevant channels and removing the others. Figure 2 represents the identified relevant electrodes for each finger in all subjects. As depicted in Figure 2, despiteusing 64 channels during the acquisition process, more than 70% of the channels were removed due to not containing useful information. Furthermore, the most sensitive channels were in the left hemisphere and the center of the cortex as the scope of this study focused on the movements of fingers of the right hand. These results were obtained with an α_threshold set to 70%.

**Algorithm 1:** Commented algorithm of the basic steps of feature extraction and classification problems

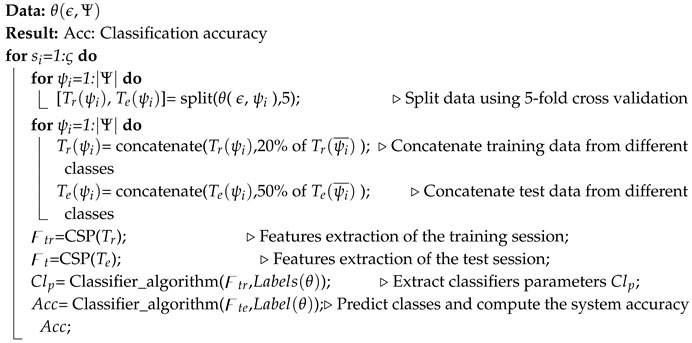



**Figure 3:**
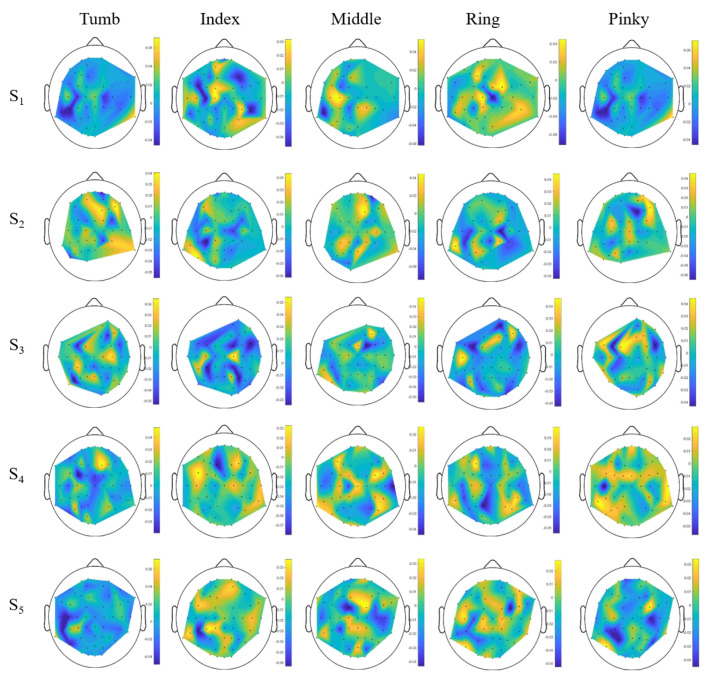
Electrode weights obtained for each finger and each subject.

Figure 3 shows the spatial filters, obtained with CSP algorithm after identification of relevant electrodes. The obtained features are available online at Supplementary Materials (Folder S2). These maps show that the location of relevant electrodes changes from one subject to another and from one finger to another for the same subject, as expected from the literature [26]. The features are generally smoother and physiologically more relevant, with strong weights over the motor cortex areas, except in some cases where the features have large weights in several unexpected locations from a neuro-physiological point of view.

Four different metrics have been used to measure the efficiency of the proposed method to detect fingers movements. These metrics are: the Accuracy, Precision, Recall, and the F-measure.

The integration of the channel selection algorithm with the same feature extraction as well as classification algorithms enhanced the accuracy of the system significantly, by more than 20% for each subject. The obtained system accuracy was 81% overall. Table 1a–e present the significance test for the five subjects. The column “Raw Accuracy” of these tables shows the accuracy of the classification process of finger movements using CSP and LDA without our proposed statistical based channel selection. The results demonstrate the efficiency and the validity of our approach. Table 1f presents the accuracy obtained for each subject. Despite the system classifying five finger movements simultaneously, the measured accuracy exceeded 83%, which exceeds all previous literature concerning EEG and fingers. In fact, with the use of the same approach, multiple finger movements can be detected simultaneously, in addition to having high model accuracy.

## 4. Discussion

This research was implemented with the use of a relatively large amount of data instances. The sessions of the recording will hopefully help other BCI researchers bring further improvements based on this data. The starting prediction models on the raw data ranged from 52% to 59% and this was highly improved with intensive cleaning and the addition of a pre-processing phase. Having an accuracy of 83% proves the quality of the decided approach. Our pre-processing approach showed high accuracy results by choosing the relevant channels after their comparison with reference channels and with the exclusion of unimportant channels. The prediction model will get a differentiable dataset by using this pre-processed sequence along with the feature extraction approach that can be classified using a machine learning model such as the LDA. The main advantage of giving every finger its classifier is that it allows the model to predict movements of multiple fingers at the same time, which has not been done or discussed in previous works. The determination of the flexion angle of the fingers and distinguishing between different finger movements extracted from both hands could be investigated in future works, along with improving the accuracy of the current system.

Table 2 presents the accuracy in association with the proposed system and the overall results of existing methods that are validated according to the online and offline approach. System performance is significantly improved by the proposed system, achieving a system accuracy of 81% on average according to the offline approach. Therefore, the proposed finger decoding system outperforms those described in previous studies in multiple ways. For example, the average accuracy described herein increased by 4% compared to the best known previous system that was presented in [10]. Moreover, the proposed system significantly improves the runtime using robust and efficient algorithms in contrast with the method presented in [7,8,9,10].

A small number of trials for every finger movement are sufficient to train the system for the on-line scenario before being used by a new user. Indeed, the number of training set could be increased using data augmentation techniques. The data augmentation technique which we have adopted in this work consists of decomposing every trial *t* of a total time *T* into fragments of an equal small window size *W*. Having fragments with small duration does not harm the proposed method since the features are extracted from the frequency domain of the trial and not from its time domain. Moreover, every two consecutive fragments may overlap. Let’s define *O* as the overlapping ratio. This ratio determines how much data are common between two consecutive fragments. Thus, every trial t will be split into *N* fragments of a window size *W*. *N* is calculated as follows:

(6) $$N=\frac{T-W}{shift}+1\mathrm{where}shift=\left(1-O\right)W.$$

Therefore, if we consider a trail *t* of 6 s. The trial *t* could be split into N=(6−2)/(0.25×2))+1=9 fragments of 2 s each with an overlapping ratio of 75%. Thus, five trials of 6 s will be decomposed to 45 trials of 2 s.

## 5. Conclusions

This study aimed to develop a BCI system for disabled people who are suffering from motor mobility impairment. The outcomes of this study may contribute to the development of a next-generation prosthesis, i.e., a brain-controlled prosthesis. Such prosthesis can offer an alternative method for disabled people to restore their mobility. In this study, a prediction method was developed that consists of a set of one-class classifiers. An average accuracy of 81% was achieved with the proposed method. Existing EEG BCI systems decode only single finger movements. In contrast, different models were trained during the establishment of this system, including the SVM, Gaussian, Naïve Bayes, Linear Regression, as well as the LDA model. The LDA was the classifier which obtained the best results. The system was trained and tested using a data-set recorded from volunteers. These promising results can significantly increase the control of EEG-based BCI technologies and potentially facilitate their development with rich control signals to drive complex applications. The detection of continuous finger movements will be the target of the future work associated with this system.

## Figures and Tables

**Table 1:** Subject’s statistical testing.

(a) $s_{1}$ results					
Finger	Raw Accuracy	Proposed method			
		Accuracy	Precision	Recall	F_Measure
Thumb	50	80.35	85.71	83.33	84.50
Index	60.70	80.35	90.32	77.77	83.58
Middle	57.14	83.92	86.48	88.88	87.67
Ring	55.35	85.71	91.17	86.11	88.57
Pinky	57.14	87.50	93.93	86.11	89.85
**(b)** $s_{2}$ **results**					
Finger	Raw Accuracy	Proposed method			
		Accuracy	Precision	Recall	F_Measure
Thumb	66.07	73.21	81.81	75	78.26
Index	58.18	85.45	93.54	82.85	87.87
Middle	61.81	89.09	91.42	91.42	91.42
Ring	56.36	83.63	96.42	77.14	85.71
Pinky	60.71	73.21	86.20	69.44	76.92
**(c)** $s_{3}$ **results**					
Finger	Raw Accuracy	Proposed method			
		Accuracy	Precision	Recall	F_Measure
Thumb	58.62	75.86	78.57	86.84	82.50
Index	53.44	79.31	82.50	86.84	84.61
Middle	55.17	89.65	88.09	97.36	92.50
Ring	53.44	84.48	87.17	89.47	88.31
Pinky	42.1	85.96	91.42	86.48	88.88
**(d)** $s_{4}$ **results**					
Finger	Raw Accuracy	Proposed method			
		Accuracy	Precision	Recall	F_Measure
Thumb	75	69.64	74.35	80.55	77.33
Index	60.71	85.71	85.	94.44	89.47
Middle	50	83.92	81.39	97.22	88.60
Ring	56.36	72.72	72.72	91.42	81.01
Pinky	56.36	78.18	78.04	91.42	84.21
**(e)** $s_{5}$ **results**					
Finger	Raw Accuracy	Proposed method			
		Accuracy	Precision	Recall	F_Measure
Thumb	51.78	76.78	81.08	83.33	82.19
Index	60.71	83.92	82.92	94.44	88.31
Middle	58.92	76.78	75.55	94.44	83.95
Ring	67.85	78.57	76.08	97.22	85.36
Pinky	60.71	76.78	76.74	91.66	83.54
**(f) Summary of the accuracy by subject**					
Finger	$s_{1}$	$s_{2}$	$s_{3}$	$s_{4}$	$s_{5}$
Thumb	80.35	73.21	75.86	69.64	76.78
Index	80.35	85.45	79.31	85.71	83.92
Middle	83.92	89.09	89.65	83.92	76.78
Ring	85.71	83.63	84.48	72.72	78.57
Pinky	87.50	73.21	85.96	78.18	76.78
Average	83.56	80.91	83.52	78.03	78.56

**Table 2:** Comparison of the proposed method system with other EEG finger decoding systems.

Studies	N${}^{\circ }$ of Fingers	Signal Processing Chain	N${}^{\circ }$ of Subjects	Accuracy (%)
[7]	2	Band-pass filter & ERD/ERS & SVM	10	≈62.5
[8]	4	CWD & 2LCF	18	43.5
[9]	5	RF & LDA & SVM & KNN	4	54
[10]	5	PCA & PSD & SVM	11	77
Proposed method	5	Band-pass filter & CAR &CSP & LDA	5	81

## Supplementary Materials

The following are available online at https://zenodo.org/record/4316450#.X9MdDrMRWUk. Folder S1: Power spectrum of each finger trial for every subject obtained by applying the FFT on the temporal signals. Folder S2: CSP weight filter of each trial for every subject obtained by applying the CSP on the corresponding relevant electrodes.

## Abbreviations

The following abbreviations are used in this manuscript:

MDPI	Multidisciplinary Digital Publishing Institute
DOAJ	Directory of open access journals
TLA	Three letter acronym
BCI	Brain Computer Interfaces
SVM	Support vector machine
LDA	Linear discriminant analysis
RF	random forest
kNN	k-nearest neighborhood
HCI	Human computer interaction
EEG	Electroencephalogram
MEG	Magnetoencephalography
ECoG	Electrocorticogram
fNIRS	Functional Near-Infrared Spectroscopy
EMG	Electromyography
UN	United Nations
ERPs	Event-related potentials
SMR	sensorimotor rhythms
CWD	Choi–Williams distribution
2LCF	Two-layer classification framework
CSP	Common spatial pattern
LSTM	long short-term memory
CNN	convolutional neural network model
RCNN	recurrent convolutional neural network
PCA	Principal component analysis
PSD	Power Spectral Densities
SNR	signal-to-noise ratio
SPI	Scenario program interface
